# Editorial: GPER and Human Pathologies

**DOI:** 10.3389/fendo.2021.794332

**Published:** 2021-11-10

**Authors:** Yves Jacquot, Marilena Kampa, Sarah H. Lindsey

**Affiliations:** ^1^ CiTCoM, CNRS UMR 8038, INSERM U1268, Faculté de Pharmacie de Paris, Université de Paris, Paris, France; ^2^ Laboratory of Experimental Endocrinology, School of Medicine, University of Crete, Heraklion, Greece; ^3^ Department of Pharmacology, Tulane University, New Orleans, LA, United States

**Keywords:** G protein-coupled estrogen receptor, human pathologies, cancer, inflammation, nociception, brain, cardiovascular and digestive systems, metabolism

More than 40% of commercialized drugs exert their action through G protein-coupled receptors (GPCRs), indicating that the modulation of these hepta-transmembrane proteins is important for the control of downstream signaling pathways and related diseases. Twenty-five years ago, a new orphan GPCR initially named CMKRL2 was discovered by Owman et al. (1). This receptor was named CMKRL2 as it was shown high degree of identity and consensus features with chemoattractant receptors, in activated B cells isolated from Burkitt's lymphoma (1). Later, it was characterized by Carmeci et al. in breast cancer cells and claimed to participate in hormonal response (2). Thus, it was renamed GPR30 and, later, GPER (for G protein-coupled estrogen receptor), as it was demonstrated to bind estradiol. Since then, its role in different physiological systems and diseases has been revealed, particularly in metabolic disorders, cancer, immunity and inflammation, cardiovascular function as well as the brain. Its role in metabolic disease and cancer is highlighted by Rouhimoghadam et al. In connection with the second point, implications of GPER for anti-estrogen therapy are indisputable. In addition, epidemiologic studies indicate its potential as a valuable prognostic factor, particularly in the context of cancer. For example, high expression of GPER and DKK2 correlates with survival in epithelial ovarian cancer, as demonstrated by Fraungruber et al. The fact that the first peptidic GPER modulator ERα17p, an inverse agonist which has been designed from estrogen receptor α (3), exerts both anti-proliferative (4) and anti-nociceptive actions at similar *in vivo* doses, reflects not only the multifaceted character of GPER but also the possibility to simultaneously treat through a same target two or more pathologies (Mallet et al.).

In the light of the above considerations, Kim and Jung propose that interfering with GPER could open new avenues for the development of original therapeutic approaches devoted to the control of tumor growth. These authors have demonstrated that chrysin-nanoparticles inhibit the growth of triple negative breast tumors and associated metastasis in xenografted mice. Even if GPER signaling is predominantly estradiol-dependent, its control could be extended also to males with testicular germ cell cancers, as discussed by Chevalier et al. In fact, GPER is expressed in most malignant diseases such as melanoma, breast, pancreatic, prostate, colorectal and hepatocellular cancers. It shares also important functions in immune responses, as clearly stated by Notas et al., with a role in autoimmune pathologies such as multiple sclerosis, Parkinson’s disease and atherosclerosis-related inflammation. Hernández-Silva et al. demonstrate that GPER interferes in the development and immune response in female reproductive cancers. In this regard, it should be stressed that the GPER inverse agonist ERα17p shares, additionally to its antiproliferative and anti-nociceptive profiles, anti-inflammatory action (see Mallet et al.). These observations are closely related to the expression of GPER in B and T lymphocytes, monocytes, eosinophils and neutrophils. In the cardiovascular compartment, chronic activation of GPER protects against oxidative stress-induced cardiomyoblast death (Imam Aliagan et al.). Also present in the digestive system, GPER could be of interest in the control of the gallstone formation, a major hepatobiliary disease with a higher prevalence in women than in men, or of irritable bowel syndrome, inflammatory bowel diseases and colorectal cancer (DeLeon et al. and Jacenik and Krajewska). The issues addressed in this Research Topic reveal a key role for GPER in a panel of pathologies. Even if the number of GPER modulators is limited, its control could undoubtedly open new exciting medical approaches.

## Author Contributions

All authors listed have made a substantial, direct, and intellectual contribution to the work and approved it for publication.

## Funding

This work was supported by National Institutes of Health grant number HL133619 and the German Academic Exchange Service (DAAD project-ID: 57515112).

## Conflict of Interest

The authors declare that the research was conducted in the absence of any commercial or financial relationships that could be construed as a potential conflict of interest.

## Publisher’s Note

All claims expressed in this article are solely those of the authors and do not necessarily represent those of their affiliated organizations, or those of the publisher, the editors and the reviewers. Any product that may be evaluated in this article, or claim that may be made by its manufacturer, is not guaranteed or endorsed by the publisher.
